# Supplementary material for: Ducks change wintering patterns due to changing climate in the important wintering waters of the Odra River Estuary
Source: PeerJ. 2017 Jul 31;5:e3604. doi: 10.7717/peerj.3604 (PMC5541925; doi:10.7717/peerj.3604)
Supplement: Table S2 — The models were ranked using the Akaike information criterion (AIC). Δ AIC represents the difference between each model and the best-fit model. wi –Akaike weight (indicating model probabilities); df, –degrees of freedom. The terms in the models are represented by numbers: 1 –feed, 2 –ice cover, 3 –max ice, 4 –season, 5 –ice cover*feed, 6 –max ice*feed, 7 –season*feed. [file peerj-05-3604-s002.docx]

**Supplementary material**

Table S2. Ranking of general linear mixed models showing the influence of ice cover, maximum ice extent [km^2^] in the Baltic Sea (max ice) and season on the percentages of the population of the target species in the Odra River Estuary. The models were ranked using the Akaike information criterion (AIC). ΔAIC represents the difference between each model and the best-fit model. wi – Akaike weight (indicating model probabilities); df, – degrees of freedom. The terms in the models are represented by numbers: 1 –feed, 2 – ice cover, 3 – max ice, 4 – season, 5 – ice cover*feed, 6 – max ice*feed, 7 – season*feed.

| Model | df | AICc | ΔAIC | wi |
| --- | --- | --- | --- | --- |
| 1234567 | 12 | 104.59 | 0 | 0.7 |
| 123457 | 11 | 107.5 | 2.9 | 0.16 |
| 12457 | 10 | 108.45 | 3.86 | 0.1 |
| 12356 | 10 | 112.21 | 7.62 | 0.02 |
| 1235 | 9 | 114.06 | 9.47 | 0.01 |
| 123456 | 11 | 114.26 | 9.66 | 0.01 |
| 125 | 8 | 115.7 | 11.11 | 0 |
| 12345 | 10 | 116.09 | 11.5 | 0 |
| 1245 | 9 | 117.01 | 12.42 | 0 |
| 12347 | 10 | 145.96 | 41.37 | 0 |
| 123467 | 11 | 146.7 | 42.11 | 0 |
| 1247 | 9 | 146.73 | 42.14 | 0 |
| 123 | 8 | 157.78 | 53.19 | 0 |
| 23 | 7 | 158.08 | 53.49 | 0 |
| 12 | 7 | 159.01 | 54.42 | 0 |
| 1236 | 9 | 159.24 | 54.65 | 0 |
| 2 | 6 | 159.33 | 54.74 | 0 |
| 1234 | 9 | 159.86 | 55.27 | 0 |
| 234 | 8 | 160.14 | 55.55 | 0 |
| 124 | 8 | 160.58 | 55.98 | 0 |
| 24 | 7 | 160.85 | 56.26 | 0 |
| 12346 | 10 | 161.33 | 56.74 | 0 |
| 1347 | 9 | 162.96 | 58.37 | 0 |
| 13467 | 10 | 163.76 | 59.17 | 0 |
| 147 | 8 | 167.7 | 63.11 | 0 |
| 13 | 7 | 174.33 | 69.73 | 0 |
| 3 | 6 | 174.61 | 70.02 | 0 |
| 136 | 8 | 175.8 | 71.21 | 0 |
| 134 | 8 | 176.03 | 71.44 | 0 |
| 34 | 7 | 176.28 | 71.69 | 0 |
| 1346 | 9 | 177.52 | 72.93 | 0 |
| 14 | 7 | 180.48 | 75.89 | 0 |
| 4 | 6 | 180.71 | 76.11 | 0 |
| 1 | 6 | 181.15 | 76.56 | 0 |
| Null | 5 | 181.45 | 76.86 | 0 |
