# Supplementary material for: Ducks change wintering patterns due to changing climate in the important wintering waters of the Odra River Estuary
Source: PeerJ. 2017 Jul 31;5:e3604. doi: 10.7717/peerj.3604 (PMC5541925; doi:10.7717/peerj.3604)
Supplement: Table S3 — The parameters show the interaction between season and species, ice cover and species, max ice and species. The interaction parameters species*season, species*ice cover, species*max ice were used to predict the values presented in Fig. 2. [file peerj-05-3604-s003.docx]

**Supplementary material**

Table S3. Results of general linear mixed models showing the influence of ice cover, maximum ice extent [km^2^] in the Baltic Sea (max ice) and season on the percentages of the population of the target species in the Odra River Estuary. The parameters show the interaction between season and species, ice cover and species, max ice and species. The interaction parameters species*season, species*ice cover, species*max ice were used to predict the values presented in Figure 2.

| Model Term | Coefficient | Std. Error | t | *P* |
| --- | --- | --- | --- | --- |
| Intercept | -15.447 | 13.901 |  |  |
| Ice cover | -0.039 | 0.007 | -5.273 | **<0.001** |
| Season | 0.008 | 0.007 | 1.158 | 0.248 |
| Max ice | <0.001 | 0.001 | 0.325 | 0.745 |
| spec=Coot | 13.350 | 18.149 | 0.736 | 0.463 |
| spec=Goldeneye | 14.916 | 18.149 | 0.822 | 0.412 |
| spec=Goosander | 30.270 | 18.149 | 1.668 | 0.096 |
| spec=Pochard | 29.661 | 18.149 | 1.634 | 0.103 |
| spec=Scaup | -45.932 | 18.149 | -2.531 | **0.012** |
| spec=Smew | 52.031 | 18.149 | 2.867 | **0.004** |
| spec=Tufted |  |  |  |  |
| Season*[spec= Coot] | -0.007 | 0.009 | -0.749 | 0.455 |
| Season*[spec=Goldeneye] | -0.007 | 0.009 | -0.830 | 0.407 |
| Season*[spec=Goosander] | -0.015 | 0.009 | -1.654 | 0.099 |
| Season*[spec= Pochard] | -0.015 | 0.009 | -1.650 | 0.100 |
| Season*[spec=Scaup] | 0.023 | 0.009 | 2.553 | **0.011** |
| Season*[spec= Smew] | -0.026 | 0.009 | -2.849 | **0.005** |
| Season*[spec=Tufted] |  |  |  |  |
| Ice cover*[spec=Coot] | 0.024 | 0.010 | 2.386 | **0.018** |
| Ice cover*[spec=Goldeneye] | 0.019 | 0.010 | 1.931 | 0.054 |
| Ice cover*[spec=Goosander] | 0.071 | 0.010 | 7.118 | **<0.001** |
| Ice cover*[spec=Pochard] | 0.025 | 0.010 | 2.539 | **0.012** |
| Ice cover*[spec=Scaup] | -0.026 | 0.010 | -2.592 | **0.010** |
| Ice cover*[spec= Smew] | 0.033 | 0.010 | 3.315 | **0.001** |
| Ice cover*[spec=Tufted] |  |  |  |  |
| Max ice*[spec= Coot] | -0.091 | 0.069 | -1.313 | 0.190 |
| Max ice*[spec=Goldeneye] | -0.063 | 0.069 | -0.912 | 0.363 |
| Max ice*[spec=Goosander] | -0.142 | 0.069 | -2.055 | **0.041** |
| Max ice*[spec= Pochard] | -0.088 | 0.069 | -1.264 | 0.207 |
| Max ice*[spec=Scaup] | 0.071 | 0.069 | 1.027 | 0.305 |
| Max ice*[spec=Smew] | -0.115 | 0.069 | -1.658 | 0.098 |
| Max ice*[spec=Tufted] |  |  |  |  |
| method (r) | 0.001 | 0.002 |  |  |
| month (r) | 0.016 | 0.020 |  |  |
