# Supplementary material for: Ducks change wintering patterns due to changing climate in the important wintering waters of the Odra River Estuary
Source: PeerJ. 2017 Jul 31;5:e3604. doi: 10.7717/peerj.3604 (PMC5541925; doi:10.7717/peerj.3604)
Supplement: Table S4 — (1) Target species, (2) trend of flyway population after Nagy, Flink & Langendoen, 2014; (3) direction of population index change in the ORE; (4) impact of ice cover in the ORE on the dependent variable; (5) impact of ice cover in the whole Baltic on the dependent variable. [file peerj-05-3604-s004.docx]

**Supplementary materials**

Table S4. Trend of flyway population (2) and impact of covariates (3-5) on the dependent variable – the ratio of the percentage of the numbers of a given species in the study area to the estimated total flyway population in a given year. (1) Target species, (2) trend of flyway population after Nagy et al. 2014; (3) direction of population index change in the ORE; (4) impact of ice cover in the ORE on the dependent variable; (5) impact of ice cover in the whole Baltic on the dependent variable.

| Target species (1) | Trend of flyway population (2) | Index trend in the ORE (3) | Impact of ice cover in the ORE (4) | Impact of ice cover in the whole Baltic (5) |
| --- | --- | --- | --- | --- |
| Greater Scaup | ↓ | ↑ | ↓ | → |
| Tufted Duck | ↓ | ↑ | ↓ | → |
| Common Pochard | ↓ | ↓ | ↓ | ↓ |
| Common Goldeneye | ↑ | → | ↓ | → |
| Eurasian Coot | → | → | ↓ | ↓ |
| Smew | ↑ | ↓ | → | → |
| Goosander | → | → | → | → |
